# Real Time PCR-based diagnosis of human visceral leishmaniasis using urine samples

**DOI:** 10.1371/journal.pgph.0000834

**Published:** 2022-12-29

**Authors:** Samiur Rahim, Md. Mohiuddin Sharif, Md. Robed Amin, Mohammad Tariqur Rahman, Muhammad Manjurul Karim

**Affiliations:** 1 Department of Microbiology, University of Dhaka, Dhaka, Bangladesh; 2 Department of Medicine, Dhaka Medical College, Dhaka, Bangladesh; 3 Faculty of Dentistry, University of Malaya, Kuala Lumpur, Malaysia; Banaras Hindu University, INDIA

## Abstract

Diagnosis of visceral leishmaniasis (VL) through the detection of its causative agents namely *Leishmania donovani* and *L*. *infantum* is traditionally based on immunochromatographic tests, microscopy of bone marrow, spleen aspirates, liver or lymph node and differential diagnosis. While the first process has low specificity, the later one carries the risk of fatal hemorrhage. Over the last decade, multiple Polymerase Chain Reaction (PCR) based diagnosis has been developed using blood and urine sample with a varying degree of sensitivity and specificity, an issue worth improving for precision diagnosis. Earlier, we reported a PCR-based diagnosis of *L*. *donovani* in peripheral blood using a novel set of PCR primers with absolute specificity. Using the same set of primers and PCR conditions, here we describe diagnosis of *L*. *donovani* from urine, for a non-invasive, rapid and safe diagnosis. Diagnosis of *VL* was carried out using urine samples collected from clinically diagnosed VL patients (n = 23) of Bangladesh in Real Time PCR. Test results were validated by comparing blood samples from the same set of patients. Sensitivity and specificity of this diagnosis was analyzed using retrospective bone marrow samples, collected earlier from confirmed VL patients (n = 19). The method showed 100% sensitivity in detecting L. *donovani* in urine and corresponding blood and retrospective bone marrow samples, as well as 100% specificity in control groups. A Real Time PCR-based molecular detection system using urine sample is hereafter presented what could be a, non-invasive approach for VL detection with precision and perfection.

## Introduction

The protozoan parasite *Leishmania donovani* (LD) is the causative agent for visceral leishmaniasis (VL) or kala-azar (KA), which is a vector-borne disease transmitted by the bite of female sand fly. The parasite exists in two stages to complete its life cycle: Amastigote (developed in human) and Promastigote (in sand fly). The amastigote form residing in the cells of mononuclear phagocytic system (MPS), like macrophage, neutrophil and endothelial cells of human, multiplies by binary fission until the cells of the MPS enlarged. Infected cells are eventually ruptured and the parasites are liberated in the circulation and invade fresh cells; the cycle is repeated until all cells of MPS are affected. Proliferation of MPS cells leads to massive splenomegaly and hepatomegaly. The bone marrow is also involved, resulting in pancytopenia.

Approximately 90% of the 500,000 estimated annual cases of kala azar occur in rural areas of Bangladesh, India, Nepal, Sudan and Brazil [1] whereas Bangladesh, India and Nepal account for 60% of global cases caused by LD. This disease accounts for 40,000 deaths annually [2]. In Bangladesh, 45 out of 64 districts are endemic for VL, while 20 million people are at risk of developing VL [3].

In recent years, Polymerase Chain Reaction (PCR) as well as Real-Time PCR based diagnostic methods have been evaluated for the diagnosis of VL and shown excellent sensitivities and specificities using spleen aspirate, bone marrow, lymph node and blood [4–7]. These processes, however, are invasive and some of them require skilled health personnel to collect samples. Furthermore, possible risk of internal hemorrhage during collection of splenic aspiration or bone marrow biopsy could be lethal for patients. Urine, on the other hand is a non-invasive specimen, which could be a potential source for detection of VL, for it is a systemic and chronic disease associated with acute kidney injury (AKI).

Multiple cases of proximal tubular injury and glomerular inflammation have been reported to be related with VL associated nephropathy [8–12]. It has been postulated that glomerulonephritis occurs due to deposition of immune-complex in kidney. *Leishmania* bodies in kidney were also detected in light and electron microscopy [13], therefore it is likely to be present in urine.

Given the involvement of VL associated nephropathy, a PCR-based diagnosis of VL caused by *L*. *infantum* using urine was developed in Brazil [14, 15]. However, similar strategy is yet to be adopted in Bangladesh, India and other countries of south Asia and south-east Asia for detection of VL caused by *L*. *donovani*, a species indigenous in this region [16]. Moreover, PCR-based diagnosis of VL that has been developed using blood and urine samples showed varying degrees of sensitivity and specificity owing to the variation of the PCR techniques (conventional versus Real Time -PCR), DNA extraction method, target *Leishmania* spp as it diverges from geographical regions, and the set of primers directed to bind to the template *Leishmania* chromosomes [17]. For example, VL diagnosis using urine in conventional PCR setting showed sensitivity ranging between 25% and 92.8%, while real time PCR based diagnosis showed sensitivity from 47% up to 96.8% [14, 17–20]. Moreover, due to variation in DNA extraction methods detection limit for leishmanial DNA varies and DNA extraction using phenol chloroform showed low reproducibility [5, 21]. Earlier, this lab developed a blood-based method of diagnosing VL using a novel set of primers, MK1 primer pair specific to *L*. *donovani* that produced 98% sensitivity and 100% specificity [7]. Here, we took the advantage of using the same set of primers, as it produced profound edge of sensitivity [7], over other sets of primers published in the literature [22, 23]. The suitability of MK1 primer is hereafter justified using urine as sample from patients with VL in both conventional and real time PCR settings, thereby establishing a patient-friendly, non-invasive method of disease diagnosis.

## Materials and methods

### Ethical clearance and study group

An Ethical clearance from Dhaka Medical College Hospital (DMCH), Dhaka, Bangladesh (reference: Memo No. ERC-DMC/ECC/2021/409, dated 23 Nov 2021), and Bangladesh Medical Research Council (reference number BMRC/NREC/2013-2016/816, dated 8 May 2014) as well as formal written consents from individuals participated in this study were duly obtained at the onset of the study. For participants under 18 years of age, the formal written consent was obtained from the respective parent/ guardian.

Twenty-three patients with clinically diagnosed VL (CDVL) group were enrolled in this study; twenty-one of them were admitted in the DMCH, while the rest two were in Bangabandhu Sheikh Mujib Medical University (BSMMU). Seventeen of them were adult male, five were adult female and one was a male child. Twenty participants were enrolled as healthy control (HC) group; ten each from VL endemic and non-endemic areas. There were nineteen confirmed VL (CVL) patients, and twenty-five patients formed the disease control (DC) group with symptomatic diseases, i.e., tuberculosis (n = 10), malaria (n = 3), and dengue (n = 12) as reported earlier [7] (see the first row of Table 3).

### Clinical diagnosis of the patients

Clinical diagnosis of the patients was confirmed by the clinicians at DMCH, and BSMMU, Dhaka, Bangladesh. Patients with clinical signs such as fever, splenomegaly and hepatomegaly symptoms who were admitted in DMCH and BSMMU until March 2022 were confirmed with VL by on-site serology test (rk39-based Immunochromatographic test (ICT)), microscopy of parasitology (observation of amastigote in spleen or bone marrow aspirations), and medical examination by resident clinicians.

### Sample collection

#### Urine

First morning urine samples were collected from all CDVL patients (n = 23) and stored at 4°C before DNA extraction was done within an hour of sample collection. Briefly, 50 mL of urine were collected from each individual in a 100 mL plastic container (Falcon, USA) and were centrifuged at 12,000 *×g* for 5 minutes; then the pellet was suspended in phosphate buffer saline (PBS) for DNA extraction. Urine (n = 20) was also collected from healthy control (HC) group consisting of healthy individuals of non-endemic (Dhaka, n = 10) and endemic areas (n = 10).

#### Blood

2 ml venous blood samples were collected in K3 EDTA Tube (13x75mm, Purple Cap, 2mL) from the same set of CDVL patients (*n* = 23) from where urine samples were collected. On the other hand, venous blood was also collected from both volunteers of HC group (n = 20), and patients with symptomatic diseases similar to VL of disease control group (n = 25; 10, 3 and 12 patients diagnosed for tuberculosis, malaria and dengue respectively). Blood sample processing was conducted within an hour of collection until then samples were stored at 4°C. To separate buffy coat from whole blood, blood was centrifuged at 300 x*g* for 10 minutes. Supernatant plasma above the buffy coat layer was removed and the layers of buffy coat followed by erythrocytes underneath (to a depth of about 1 mm) were collected using a Pasteur pipette.

*Bone marrow*. Retrospective bone marrow samples collected from the CVL (n = 19) patients participated in an earlier study [7] were considered for the current analyses.

*Positive control*. *L*. *donovani* DNA, obtained from culture positive promastigote was used as the positive control.

### DNA extraction

Total DNA was extracted from urine using QIAamp^®^ DNA Mini Kit (Qiagen, Germany) following manufacturer’s instructions. In order to extract DNA from blood samples, 50μL of blood buffy coat was homogenized with 1 mL of Tween 20, nonidet P-40, NaOH, Tris pH 7.2 (TNNT) buffer and 10μL of a proteinase K solution (containing 320μL of the enzyme/mL) in a 1.5 mL micro-centrifuge tube. Then it was incubated for 3 hours at 56°C followed by an incubation at 72°C for 10 min. The homogenate was then kept at 37°C overnight before 200μL of a phenol–chloroform–isoamyl alcohol mixture (25:24:1 by volume) were added. After vigorous shaking, the mix was centrifuged (10,000 ×g for 10 min), and then the DNA in the aqueous layer was separated, and precipitated with 400μL cold absolute ethanol. The pellet was re-suspended in 50μL double-distilled water before storing at 4°C.

### Conventional PCR reaction

Five microliters of purified DNA extracted from both blood or urine samples was used as template in polymerase chain reactions (PCR). The templates were probed using the same MK1F/R primers (Forward primer 5’-CCCAAACTTTTCTGGTCCTC-3’, Reverse primer 5’-GAGCCGATTTTTGGCATTT-3’) which were designed to amplify a 102 bp fragment of leishmanial kinetoplast DNA for the detection of VL [7]. The reaction was performed with an initial denaturation step at 95°C for 10 min, followed by 40 cycles of amplification (95°C/30 sec, 45°C/30 sec, 72°C/30 sec). The PCR products were analyzed by electrophoresis on a 1.2% (w/v) agarose (Thermo Scientific, Mol Bio Grade, USA) gel, containing ethidium bromide (0.5 mg/mL in TBE buffer), 0.04 M Tris-borate and 0.001M EDTA; thereafter photographed under UV illumination using Gel documentation system (Alpha Imager HP System Versatile Gel Imaging, Santa Clara, CA, USA).

### Real-Time PCR reaction

Real-time PCR based detection of VL was conducted using the MK1F/R primers in the ABI PRISM 7000 system (Applied Biosystems, USA). For each 20μL reaction mix a 5μL sample of DNA, 1μL of 10 μmol/L of each primer MK1F and MK1R [7], 10μL GoTaq™ qPCR Master Mix (containing BRYT Green^®^ Dye having spectral properties similar to those of SYBR® Green I Promega™, USA), and 3μL of nuclease-free water were added. The reaction was performed with an initial denaturation step at 95ᴼC for 10 min, followed by 40 cycles of amplification (95°C/30 sec, 60°C/30 sec). Non-template control (NTC) and positive control were included. ABI PRISM software (version 1.1) was used for result analysis. Melt curve pattern for the PCR amplicons was produced using a temperature gradient from 60°C to 95°C. The melt pattern was analyzed for the pick at 82±2°C, the theoretical melting temperature of the 102 bp-long amplicon of MK1 primer pair.

### Sensitivity, linearity and reproducibility of Real Time PCR assay

A known six 10-fold serial dilutions of DNA (1 ng to 10fg/μL) extracted from *in vitro* cultured promastigotes of *L*. *donovani* corresponding to 10,000 to 0.1 parasites per microliter was used to determine the minimal number of parasites that could be calibrated by the assay. Each reaction contained 1μL of parasite DNA. Three replicates of six 10-fold DNA concentrations were tested in a single run for intra-assay validation. For inter assay validation, similar dilutions were made and three independent runs were performed. The coefficient of variation (CV; represented as the ratio of mean to standard deviation, SD) measures the assay’s variability. Melt curve analysis was conducted to evaluate the analytical specificity of real-time PCR products.

### Limit of detection for urine and blood samples

The possible PCR inhibitory effect of biological samples, urine and blood used for VL diagnosis, was analyzed to determine the limit of detection. 200μL of urine collected from one VL negative (in PCR based diagnosis) participant living in non-endemic area was mixed with *in vitro* cultured promastigotes of *L*. *donovani* to a concentration of 100 parasite bodies per microliter. DNA extracted from that spiked urine and three 10-fold serial dilutions of that DNA (10pg to 10fg/μL) corresponding to 100 to 0.1 parasites per microliter were used to determine the minimal number of parasites that could be in urine and assess the inhibitory effect of urine on the standard detection limit of the real time PCR assay.

In order to measure the limit of detection from blood samples, 200μL blood buffy-coat collected from the participant who contributed urine for the same analysis was mixed with *in vitro* cultured promastigotes of *L*. *donovani* to a concentration of 100 parasite bodies per microliter. The DNA was extracted from the spiked blood buffy-coat using previously described phenol chloroform method, thereafter was subjected to determination of limit of detection after making three 10-fold serial dilutions of that DNA (10pg to 10fg/μL) corresponding to 100 to 0.1 parasites per microliter by real time PCR assay (Including the primary DNA extract).

Each real time PCR reaction contained 1μL of DNA extract from either spiked urine or spiked blood buffy-coat. Each reaction was replicated for two times.

### Statistical analysis

Online tool Vassar Stats was used to calculate the statistical analyses at 95% confidence interval (CI). The sensitivity and the specificity of the primer pairs were calculated according to the following statistical formulas:

Sensitivity = [a/ (a + c)] × 100

Specificity = [d/ (b + d)] × 100

where a, b, c and d represent numbers of true positive, false positive, false negative, and true negative samples respectively.

## Result

### Clinical history of the patients

The clinically diagnosed VL patients (n = 23) were 16 to 86 years old (median 36 year). They were admitted with a history of 1 to 2.5 months (median 1.6 month) of fever before hospitalization. 72% of the patients had splenomegaly and 63% of them had hepatomegaly while all of them were reported to have anemia as well as weight loss. There was no post kala azar dermal leishmaniasis (PKDL) case in the study. Again, no patients took anti- leishmanial drug during the episode of VL. Samples were collected from CDVL patients admitted either at DMCH or BSMMU. The respective institutions used their standard protocol for the diagnosis of VL in CDVL patients depending on the condition of the patients, for example, microscopic examination of bone marrow (n = 1) and splenic aspirate (n = 3), and rk39 ICT test (n = 19) in addition to the on-site medical examination by the physicians. The subsequent Real Time PCR-based molecular detection was thereafter conducted using urine and blood of the clinically diagnosed VL patients.

On the other hand, DNA extracted from bone marrow samples (n = 19), collected from CVL patients participated in a preceding study [7] preserved in DNA bank of Fermentation and Enzyme Biotechnology Laboratory (FEBL), Department of Microbiology, University of Dhaka were taken into consideration for diagnosis by Real Time PCR.

### Conventional PCR

**Fig 1:**
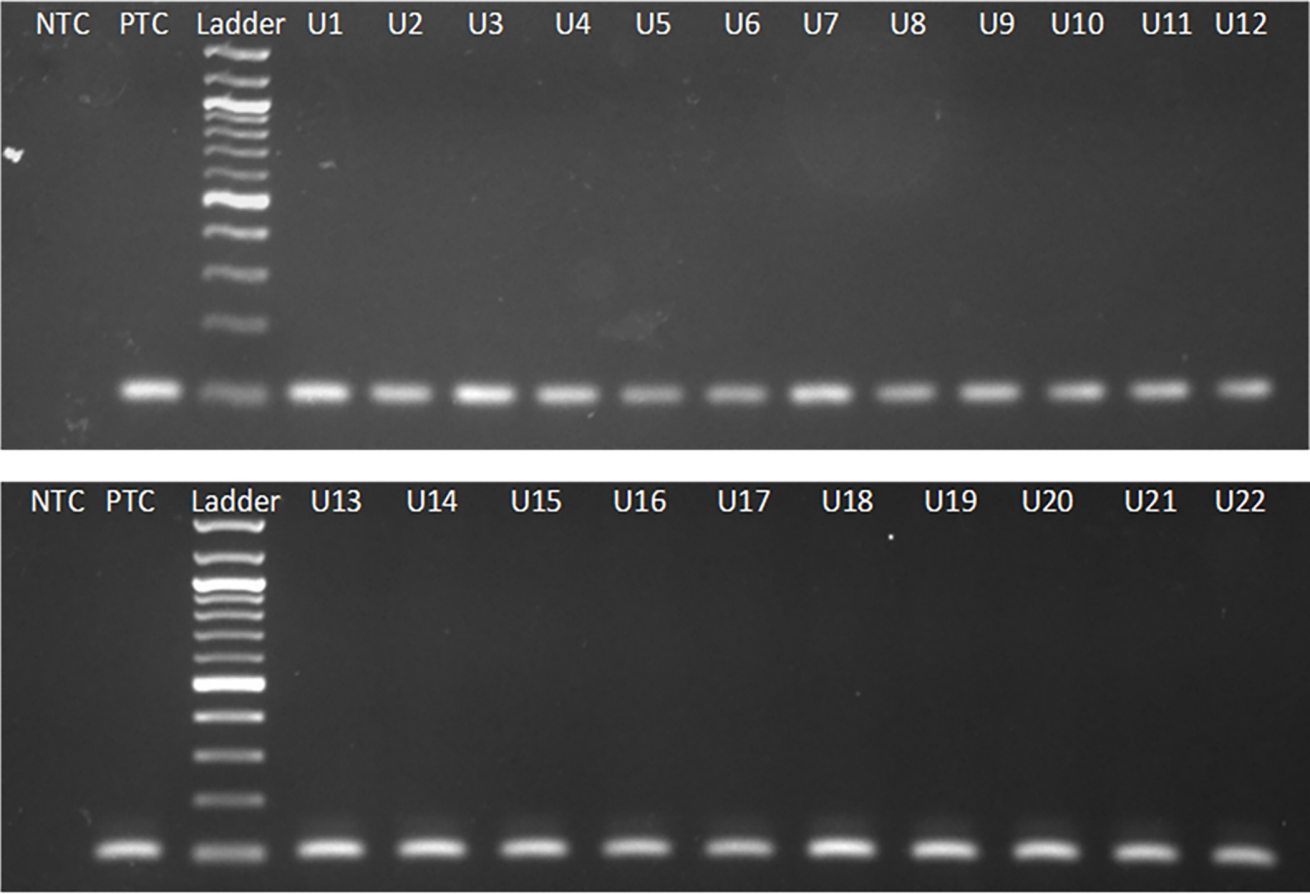
Detection of LD kinetoplast from urine of CDVL patients using novel MK1F/R primers in conventional PCR. The PCR-amplified bands using novel MK1F/R primers of 102 bp fragment of leishmanial kinetoplast DNA were produced from 22 urine samples of CDVL patients labeled U1 through U22. Amplicons were separated on a 2% agarose gel; PTC was positive template control containing DNA of cultured LD; NTC was negative template control.

DNAs collected from the urine specimens of the 23 clinically diagnosed patients, produced the amplicon of 102 bp in polymerase chain reactions when probed with MK1F/R primers (Fig 1). The finding was no different when DNAs collected from the buffy coat preparations of the corresponding patients were used as template in a similar set of PCR reaction. The diagnostic sensitivity of VL using urine as specimens was measured 100% (95% Cl, 85.19–100%) when compared to conventionally-used specimen and the peripheral blood samples (Table 3). Importantly, none of the 20 control urine samples collected from healthy individuals produced the amplicon, indicating 100% specificity of the analyses (Table 3). The success of qualitative measurement of sensitivity and specificity prompted us to estimate limits of detection of pathogen loads using quantitative Real Time PCR analyses.

### Real-Time PCR

#### Analytical sensitivity, linearity, reproducibility of Real time PCR assay

**Fig 2:**
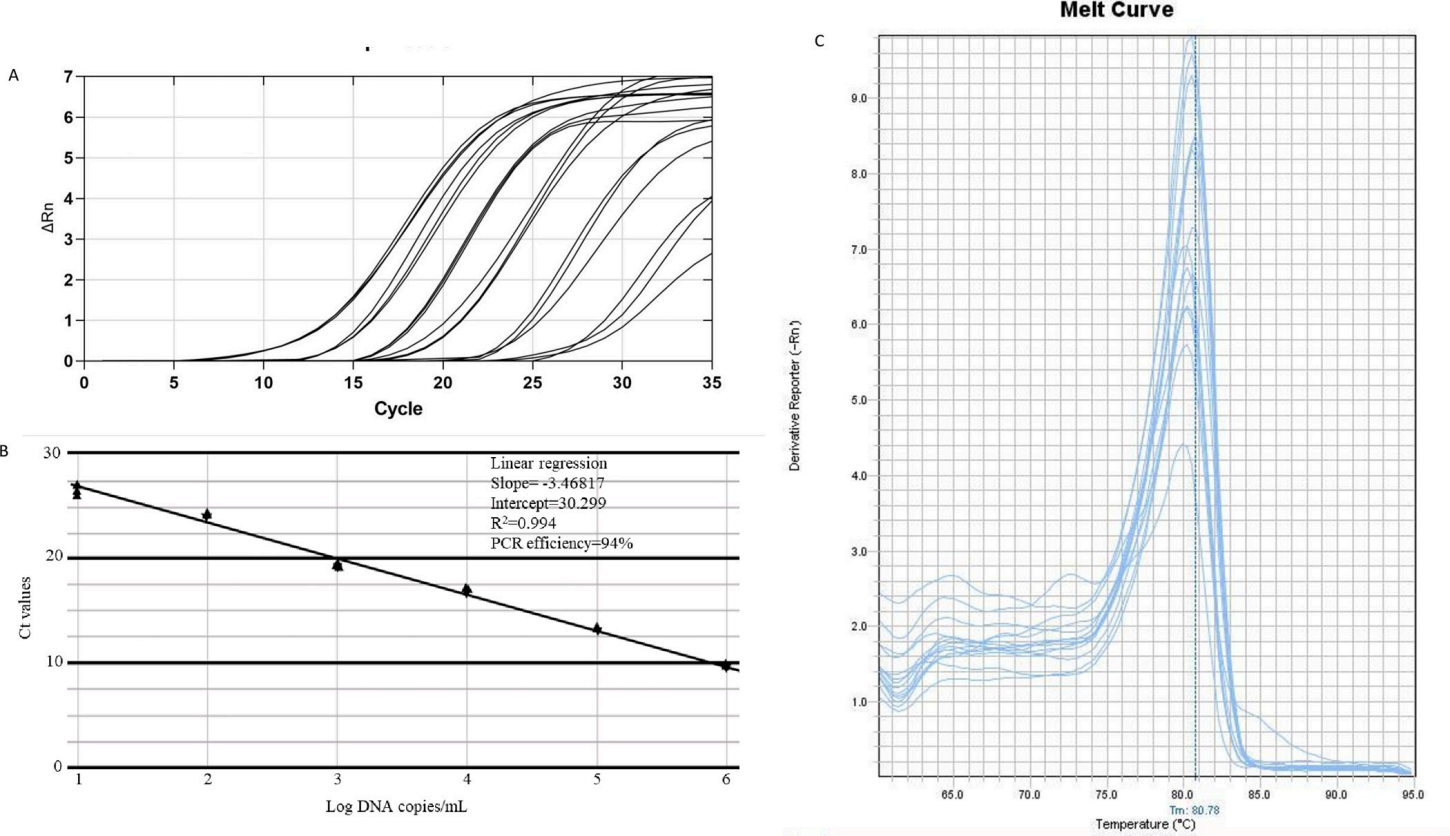
Analytical sensitivity and detection limit of VL diagnosis using Real Time PCR. Amplification curves are shown for DNA extracted from 6 log serially diluted *L*. *donovani* culture, ranging from 1x10^4^ to 1x10^-1^ parasite per microliter (A). A standard curve is constructed based on the mean of Ct values plotted against serial dilutions containing corresponding *L*. *donovani* DNA (B). Each point represents the Ct value of an individual sample and the plot represents a linear function (R^2^ = 0.994). Melt curve of amplification product showing peak near 80.78°C (C).

With discernible amplification and melt curve around 81°C, the real-time PCR assay detected as little as 10 fg of LD genomic DNA per reaction, which corresponds to 0.1 parasite (Fig 2). The standard curve produced by gradual dilution of parasite DNA over a 6-log range and cyclic threshold (Ct) values formed a linear relation, with an R^2^ of 0.994 and PCR efficiency of 94% (Fig 2B). The absence of Ct values in the non-template control validated the specific response of the reaction mix. For six distinct concentrations, the intra assay coefficient of variation (CV) of Ct values were, 0.030%, 0.057%, 0.122%, 0.166%, 0.122% and 1.140% respectively (Table 1). More variability was observed with smaller amount of parasite DNA. Inter assay variation of Ct values for the same dilution series in two independent runs was used to test the assay’s reproducibility. The coefficients of variation between assays were determined to be 1.672%, 1.645%, 2.371%, 2.576%, 1.735% and 3.4% percent, showing strong reproducibility (Table 1).

**Table 1:** Reproducibility of Real time PCR based diagnosis of VL.

Parasite Load (parasite/μL)	Intra assay Variation of Ct value (Assay 1)					Inter assay variation of Ct value			
	Replicate 1	Replicate 2	Replicate 3	Mean±SD	CV%	Assay 1	Assay 2	Mean±SD	CV%
1x10^4^	9.58	9.52	9.52	9.54±0.03	0.03%	9.54	9.23	9.38±0.157	1.67%
1x10^3^	13.44	13.43	13.42	13.43±0.008	0.058%	13.43	12.99	13.21±0.217	1.65%
1x10^2^	16.39	16.35	16.35	16.36±0.02	0.12%	16.36	17.16	16.76±0.3975	2.37%
1x10^1^	18.99	18.94	18.92	18.95±0.031	0.17%	18.95	19.95	19.45±0.501	2.57%
1x10^0^	23.9	23.98	23.91	23.93±0.029	0.1%	23.93	23.12	23.53±0.4083	1.73%
1x10^-1^	26.91	27.52	26.82	27.08±0.31	1.14%	27.08	27.99	28.03±0.953	3.4%

### Limit of detection in urine and blood sample

The limit of detection was estimated as little as 10fg DNA or 0.1 parasite per microliter of suspension in PBS. This estimate was found consistent for both urine and blood samples, collected from a single VL negative participant, spiked with *in vitro*-cultured promastigotes of *L*. *donovani* (Table 2). Furthermore, for both spiked urine and blood samples, the melt curve was consistent around 81°C. Variability was insignificant among the respective Ct values for all four dilutions of the spiked urine and blood samples (Table 2).

**Table 2:** Limit of detection of parasite body in urine and blood.

Parasite Load in urine (parasite/μL)	Intra assay Variation of Ct value		Variation of Ct value with reproducibility assay				Limit of detection
	Mean±SD	CV%	Mean Ct value from reproducibility assay	Mean Ct value from Urine spiked assay	Mean±SD	CV%	
1x10^2^	17.22±0.1	0.48%	16.76	17.22	16.99±0.23	1.35%	10fg DNA corresponding to 0.1 parasite in 1μL of urine
1x10^1^	19.81±0.09	0.43%	19.45	19.81	19.63±0.18	0.18%	
1x10^0^	24.05±0.085	0.25%	23.53	24.05	23.79±0.26	1.09%	
1x10^-1^	29.26±0.65	2.2%	28.03	29.26	28.64±0.87	3.04%	
Parasite Load in blood buffy coat (parasite/μL)	Intra assay Variation of Ct value		Variation of Ct value with reproducibility assay				Limit of detection
	Mean±SD	CV%	Mean Ct value from reproducibility assay	Mean Ct value from Blood buffy coat spiked assay	Mean±SD	CV%	
1x10^2^	16.72±0.03	0.18%	16.76	16.72	16.74±0.02	0.12%	10fg DNA corresponding to 0.1 parasite in 1μL of Blood buffy coat
1x10^1^	19.4±0.05	0.26%	19.45	19.4	19.42±0.03	0.17%	
1x10^0^	23.8±0.09	0.38%	23.53	23.80	23.67±0.14	0.60%	
1x10^-1^	27.01±0.21	0.76%	28.03	27.01	27.53±0.51	1.9%	

### Clinical sensitivity and specificity of Real time PCR based diagnosis of VL

**Fig 3:**
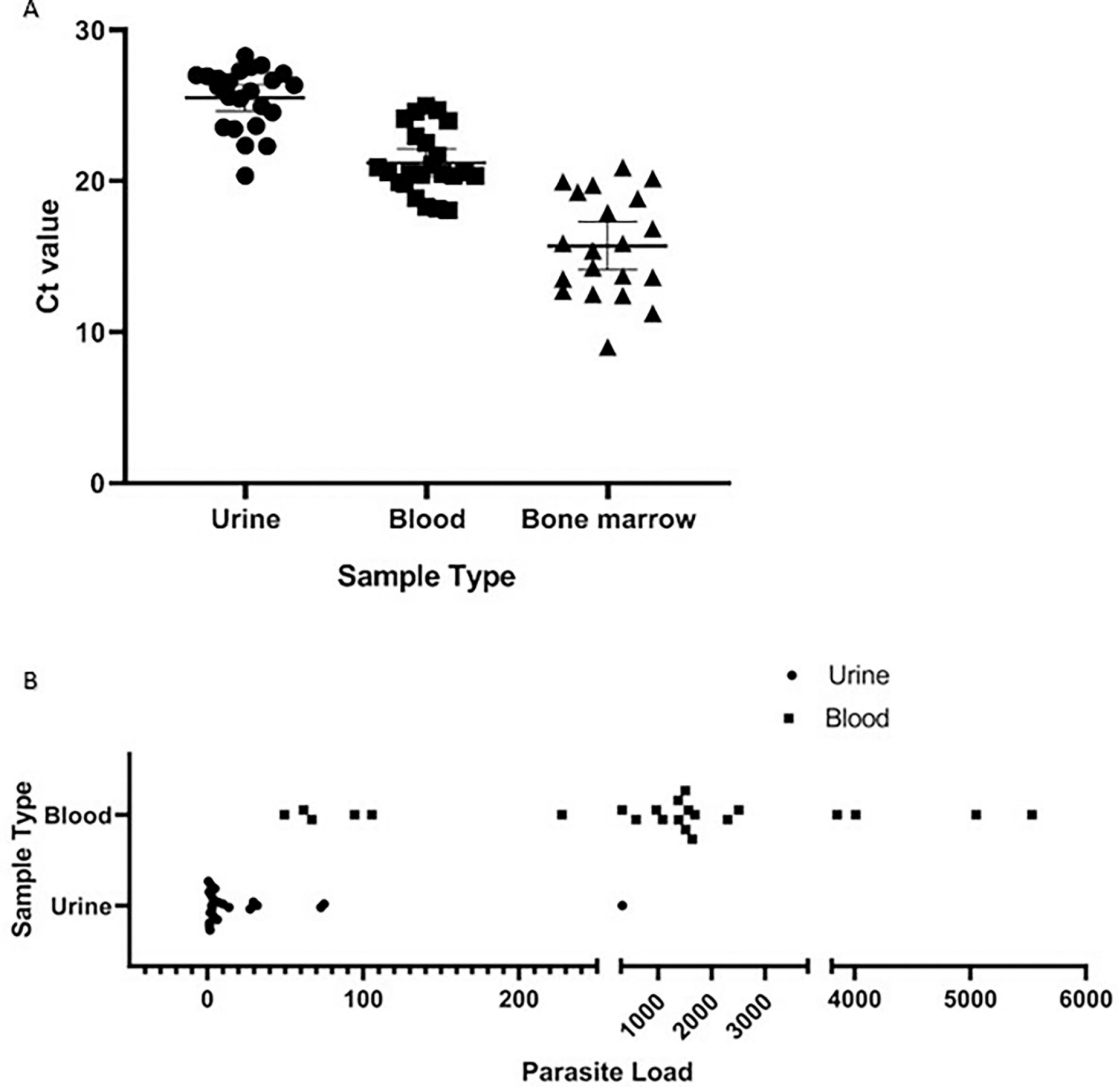
Ct value in CDVL and CVL patients and parasite load in CDVL patients. Ct value in urine from CDVL (n = 23), in blood from respective CDVL (n = 23) and in bone marrow from CVL (n = 19) patients, each point indicates data collected from individual patient (A). Parasite burden in urine and blood samples of CDVL (n = 23) patients are illustrated (B).

Presence of *L*. *donovani* was evaluated by determination of the Ct and estimation of melting temperature (Tm) using Real-time PCR method. Here, DNAs collected both from the urine of all of the 23 clinically-confirmed patients produced amplification with MK1F/R primers rendering sensitivity 100% (95% Cl, 85.19–100%) (Table 3). Detectable amplifications were found with Ct values between 15 to 28 (Fig 3A). This finding was no different when DNAs, collected from buffy coat preparations of blood specimens from corresponding patients were utilized. Retrospective DNAs extracted from bone marrow of 19 VL-confirmed patients also produced amplification with Ct values between 9 to 22, producing an absolute sensitivity, 100% (95% Cl, 82.35–100%) (Fig 3A). The detected Tm values of all types of samples was near 81°C which is close to the theoretical melting temperature of the 102 bp amplicon, 82°C, therefore validating the specificity.

**Table 3:** Comparative efficiency of Real Time PCR and conventional PCR based assays to diagnose VL using urine, blood buffy coat and bone marrow of CDVL and CVL patients.

Assay	Sensitivity (%), 95%CI			Specificity (%), 95%CI		
	CDVL (n = 23)		CVL (n = 19)	VL negative control participants (n = 45)		
	Urine (n = 23)	Blood buffy coat (n = 23)	Bone marrow (n = 19)	Urine	Blood buffy coat	
				Healthy control (n = 20; endemic control n = 10; non endemic control n = 10)	Healthy control (n = 20; endemic control n = 10; non endemic control n = 10)	Disease control (n = 25)
Real-Time PCR	23/23(100%), (85.19–100%)	23/23(100%), (85.19–100%)	19/19(100%), (82.35–100%)	0/20 (100%), (83.16–100%)	0/20 (100%), (83.16–100%)	0/25 (100%), (86.28–100%)
Conventional PCR	23/23(100%), (85.19–100%)	23/23(100%), (85.19–100%)	19/19(100%), (82.35–100%)	0/20 (100%), (83.16–100%)	0/20 (100%), (83.16–100%)	0/25 (100%), (86.28–100%)

Parasite load in two different biological samples of CDVL patients was compared and it was found that parasite load in urine, estimated 1 to 329 promastigote/mL, was comparatively lower than that of blood 49 to 5532 promastigote/mL for the same set of patients(Fig 3B).

No amplification was observed in both blood and urine samples of individuals, collected from all endemic and non-endemic healthy control with undetected Ct value in real time PCR. Again, no cross reaction was observed with DNA extracted from blood of malaria, tuberculosis and dengue patients indicating 100% (95% Cl, 82.35–100%) specificity of this diagnosis (Table 3).

## Discussion

Visceral leishmaniasis is one of the major health issues of tropical and sub-tropical countries like Bangladesh, India, Nepal, Brazil and Iran. However, rapid and accurate diagnosis of the agent is a major concern in controlling this disease. The microscopic detection of amastigote of *Leishmania donovani in* aspirates from lymph node, bone marrow and spleen are the gold standard for the diagnosis, and hold a sensitivity of 53%–65%, 53%–86% and 93%–99% respectively [24]. Although the sensitivity of the bone marrow/ splenic smear is high, collection of these specimens is not only painful but also poses a threat of fatal hemorrhage for the patients.

Alternative serodiagnosis of leishmaniasis (based on antibody detection) including ELISA showed different sensitivity and specificity depending on the type, source, and purity of the antigens. Some *Leishmania* antigens have shown cross reactivity with antigens of other microorganisms [24–26]. However, detection of antibody (IgG) in urine of patients has been used for the VL diagnosis with a sensitivity and specificity up to 98% and 95% respectively, with notable limitations: such as, false positive reactions in endemic areas, and inability to distinguish between current, subclinical, or past infections [20, 21, 27, 28].

Polymerase chain reaction (PCR)-based diagnostic methods for the detection of VL have shown excellent sensitivities and specificities using various biological samples: spleen aspirate, blood, lymph node and bone marrow [22, 29–31]. A blood-based method of diagnosis of VL using novel primer MK1F/R specific for *L*. *donovani* showed 98% sensitivity and 100% specificity [7]. Different studies showed that SYBR green Real time PCR-based diagnosis of VL targeting its minicircle DNA demonstrated sensitivity of 79% to 95.7% in blood samples [32, 33]. On the other hand, TaqMan probe based real time PCR produced up to 100% sensitivity in assays targeting genomic DNA from blood samples [34].

The use of urine as specimens has gained usefulness because of its non-invasive nature, minimal risk of blood-borne infections, and easy collection method, especially when infants are concerned. Urine-based direct anti-globulin test with the antibody (sensitivity 90.7%), and urine ELISA (sensitivity 93.3%) were reported as useful alternatives [35, 36]. PCR based diagnosis of VL using urine sample produced promising results with varying degrees of sensitivity and specificity [17]. Here, we compared a real time PCR-based diagnosis of VL caused by *L*. *donovani* using urine with that of blood and bone marrow. Primarily, the diagnosis of VL was supported by (1) the observation of amastigote in bone marrow aspirations, (2) response of rk39-ICT in serology, and (3) clinical examination by resident physicians, thereby confirming the diagnosis. In order to develop a PCR-based molecular detection method for VL that would ensure rapidity and precision of the diagnosis, urine samples of the participating patients were used as specimen; there they produced (a) bands of interest with a size of 102 bp in the electrophoretogram in conventional PCR (Fig 1) (b) the amplification with Ct values ranging 18 to 28 in a Real time -PCR (Fig 2A) after necessary extraction of DNAs. Detectable amplification was also present when analyzing the blood samples of corresponding patients, and in the retrospective 19 bone marrow samples of confirmed VL patients in the conventional PCR as well as real time PCR settings (Table 3).

In this study, Real Time PCR assay was able to detect 0.1 parasite per reaction using either urine or blood samples with a detectable melt curve. The limit of detection of this analysis is comparable and, in some cases, better than other currently available dye and probe-based Real Time PCR, while most of them were studied for VL diagnosis from blood or other more invasive biological samples [33, 34, 37, 38]. It is noticeable that the diagnosis using urine showed 100% sensitivity which is same as it showed using blood and bone marrow specimens. And this MK1(F/R) primer-based diagnosis of VL in both conventional and Real Time PCR setting using urine as well as blood showed superior sensitivity and specificity, in compared to other PCR based diagnosis of VL. In addition, this Real Time PCR assay has the potential to quantify the parasite bodies in the VL patients.

Although the target of the MK1F/R primer is the kinetoplast DNA with a tendency to vary among the parasite cells, Ct values among the replicates for particular dilutions remained consistent with very low intra-assay and inter-assay CV indicating a strong correlation between parasite load and respective Ct values (Table 2). Furthermore, the Ct values from the spiked urine and blood buffy coat also followed the earlier Ct values resulted from reproducibility assay for respective parasitic load (Table 3). The limit of detection of this real time PCR-based detection from both urine and blood was as little as 0.1 parasite body per microliter sample. Among the VL patients, parasite load was significantly higher in blood than the corresponding urine samples of the patients (Fig 3B). This comparatively low parasite load in urine was due to the fact that renal filtration system could be compromised due to immunological reactions that lead discharge of parasites or infected macrophages harboring parasites in urine [8, 9, 12, 15]. Contrarily, leishmanial promastigote infects the macrophages (in liver and spleen) and Kupffer cells (in liver) and multiply there that are eventually released in blood making their presence more prominent in blood than that of urine [39].

The road map of World Health Organization (WHO) for neglected tropical diseases (NTD) aims to eradicate VL including PKDL from 75 endemic countries by 2030 by emphasizing (a) research on the relationship among the human, vector, parasite and reservoir, (b) development of more specific and user-friendly diagnostic test, and (c) introduction of faster, more effective, safer, cost effective treatment for the VL patients worldwide [40]. Due to its commendable sensitivity and specificity for urine samples, Real-Time PCR-based diagnosis of VL using MK1 primer can be a suitable detection method to replace blood or more invasive specimens such as bone marrow or spleen. Furthermore, when compared to conventional and nested PCR-based techniques, the ease of one-step molecular detection of RT-PCR reduces work load for the pathologists, thereby fulfilling the patient-friendly diagnostic requirement to achieve the WHO objective. This method can also be used in assessing the response to therapy, monitoring parasite kinetics, infection dynamics and epidemiological survey.

The cohort of this study for diagnosis of VL from urine samples was small (n = 23), therefore further study is necessary to ascertain the degree of sensitivity and specificity of the diagnostic procedure. Moreover, the average admittance of the studied patients in the hospital was 1.7 months after the onset of illness (range 1 to 2.5 months). Therefore, the length of this diagnostic strategy beyond 2.5 months’ post-infection needs to be evaluated. Nevertheless, this is the first report of its kind in Indian sub-continent to use urine as a clinical specimen for detection of *L*. *donovani* and its impact in global public health in eradicating this NTD is significant.

## Conclusion

Invasive specimens like bone marrow, splenic aspiration or blood could be substituted by non-invasive specimen, urine to score the infection with *L*. *donovani* in patients by Real Time PCR without compromising the diagnostic efficiency of VL in Bangladesh, and this success is worth investigating in other VL endemic areas of the world.

## Supporting information

S1 Fig Raw images of Fig 1, Detection of LD kinetoplast from urine of CDVL patients using novel MK1F/R primers in conventional PCR. (PDF)

S1 Table Reproducibility of Real time PCR based diagnosis of VL (Assay 2). (DOCX)

S2 Table Limit of detection of parasite body in urine and blood. (DOCX)

S3 Table Result of conventional-PCR and Real time PCR based diagnosis of VL using urine DNA of CDVL patients. (DOCX)

S4 Table Result of conventional-PCR and Real time PCR based diagnosis of VL using blood buffy coat DNA of CDVL patients. (DOCX)

S5 Table Result of Real time PCR bone marrow DNA of CVL patients. (DOCX)

S6 Table Result of conventional-PCR and Real time PCR based diagnosis of VL using DNA of control participants. (DOCX)

## References

[1] Alvar J, Vélez ID, Bern C, Herrero M, Desjeux P, Cano J, et al. Leishmaniasis worldwide and global estimates of its incidence. PLoS One. 2012;7(5). doi: 10.1371/journal.pone.0035671 22693548 PMC3365071

[2] Ready P. Epidemiology of visceral leishmaniasis. Clin Epidemiol. 2014 May 3;6:147–54. doi: 10.2147/CLEP.S44267 24833919 PMC4014360

[3] Bhowmick A, Khanum H. Prevalence of visceral leishmaniasis, risk factors and associated disorders: Knowledge of inhabitants and professionals in Fulbaria, Mymensingh. Bangladesh J Zool. 2017 Oct 8;45:73.

[4] Mary C, Faraut F, Lascombe L, Dumon H. Quantification of Leishmania infantum DNA by a Real-Time PCR Assay with High Sensitivity. J Clin Microbiol. 2004 Dec 1;42:5249–55. doi: 10.1128/JCM.42.11.5249-5255.2004 15528722 PMC525214

[5] Pessoa-e-Silva R, Mendonça Trajano-Silva LA, Lopes da Silva MA, da Cunha Gonçalves-de-Albuquerque S, de Goes TC, Silva de Morais RC, et al. Evaluation of urine for Leishmania infantum DNA detection by real-time quantitative PCR. J Microbiol Methods [Internet]. 2016;131:34–41. Available from: doi: 10.1016/j.mimet.2016.10.002 27713020

[6] Singh OP, Sundar S. Developments in Diagnosis of Visceral Leishmaniasis in the Elimination Era. J Parasitol Res. 2015 Dec 30;2015:1–10. doi: 10.1155/2015/239469 26843964 PMC4710934

[7] Khatun M, Alam SMS, Khan AH, Hossain MA, Haq JA, Alam Jilani MS, et al. Novel PCR primers to diagnose visceral leishmaniasis using peripheral blood, spleen or bone marrow aspirates. Asian Pac J Trop Med [Internet]. 2017;10(8):753–9. Available from: doi: 10.1016/j.apjtm.2017.08.002 28942823

[8] Clementi A, Battaglia G, Floris M, Castellino P, Ronco C, Cruz DN. Renal involvement in leishmaniasis: A review of the literature. NDT Plus. 2011;4(3):147–52. 25984144 10.1093/ndtplus/sfr008 PMC4421603

[9] Verma N, Lal CS, Rabidas V, Pandey K, Singh D, Kumar S, et al. Microalbuminuria and glomerular filtration rate in paediatric visceral leishmaniasis. Biomed Res Int. 2013;2013(June). doi: 10.1155/2013/498918 23865054 PMC3705886

[10] Oliveira MJC, Silva Junior GB, Sampaio AM, Montenegro BL, Alves MP, Henn GAL, et al. Short report: Preliminary study on tubuloglomerular dysfunction and evidence of renal inflammation in patients with Visceral leishmaniasis. Am J Trop Med Hyg. 2014;91(5):908–11. 25114011 10.4269/ajtmh.14-0167 PMC4228885

[11] da Silva Junior GB, Guardão Barros EJ, De Francesco Daher E. Kidney involvement in leishmaniasis-A review. Brazilian J Infect Dis [Internet]. 2014;18(4):434–40. Available from: 10.1016/j.bjid.2013.11.013 PMC9427481 24690431

[12] Meneses GC, De Francesco Daher E, da Silva Junior GB, Bezerra GF, da Rocha TP, de Azevedo IEP, et al. Visceral leishmaniasis-associated nephropathy in hospitalised Brazilian patients: new insights based on kidney injury biomarkers. Trop Med Int Heal. 2018;23(10):1046–57. doi: 10.1111/tmi.13127 29987885

[13] Kumar R, Nylén S. Immunobiology of visceral leishmaniasis. Front Immunol. 2012;3(AUG):1–10. doi: 10.3389/fimmu.2012.00251 22912637 PMC3418610

[14] Motazedian M, Fakhar M, Motazedian MH, Hatam G, Mikaeili F. A urine-based polymerase chain reaction method for the diagnosis of visceral leishmaniasis in immunocompetent patients. Diagn Microbiol Infect Dis. 2008;60(2):151–4. doi: 10.1016/j.diagmicrobio.2007.09.001 17931819

[15] Molina I, Fisa R, Gállego M, Riera C, Portús M, Falcó V, et al. Leishmania infantum DNA Detection in Urine from Patients with Visceral Leishmaniasis and after Treatment Control. Am J Trop Med Hyg. 2018;78(5):741–4. 18458307

[16] Akter S, Alam MZ, Nakao R, Yasin MG, Kato H, Katakura K. Molecular and serological evidence of leishmania infection in stray dogs from visceral leishmaniasis-endemic areas of Bangladesh. Am J Trop Med Hyg. 2016;95(4):795–9. doi: 10.4269/ajtmh.16-0151 27382083 PMC5062775

[17] Bezerra GSN, Barbosa WL, Silva ED da, Leal NC, Medeiros ZM de. Urine as a promising sample for Leishmania DNA extraction in the diagnosis of visceral leishmaniasis–a review. Brazilian J Infect Dis [Internet]. 2019;(x x):1–10. Available from: https://linkinghub.elsevier.com/retrieve/pii/S1413867019300479 doi: 10.1016/j.bjid.2019.04.001 31054271 PMC9425670

[18] Almerice M, Medeiros Z, Regina C, Soares P, Dionísio E, Miranda-filho DB, et al. A comparison of four DNA extraction protocols for the analysis of urine from patients with visceral leishmaniasis. Rev Soc Bras Med Trop. 2014;47(April):193–7. 24861293 10.1590/0037-8682-0233-2013

[19] Mirzaei A, Ahmadipour F, Cannet A, Marty P, Delaunay P, Perrin P, et al. Immunodetection and molecular determination of visceral and cutaneous Leishmania infection using patients’ urine. Infect Genet Evol [Internet]. 2018;63(May):257–68. Available from: doi: 10.1016/j.meegid.2018.05.021 29847780

[20] Pappa SA, Kontou PI, Bagos PG, Braliou GG. Urine-based molecular diagnostic tests for leishmaniasis infection in human and canine populations: A meta-analysis. Pathogens. 2021;10(3):1–14. doi: 10.3390/pathogens10030269 33673416 PMC7996766

[21] Schijman AG, Bisio M, Orellana L, Sued M, Duffy T, Mejia Jaramillo AM, et al. International study to evaluate PCR methods for detection of Trypanosoma cruzi DNA in blood samples from Chagas disease patients. PLoS Negl Trop Dis. 2011;5(1). 10.1371/journal.pntd.0000931 PMC3019106 21264349

[22] Maurya R, Singh RK, Kumar B, Salotra P, Rai M, Sundar S. Evaluation of PCR for diagnosis of Indian kala-azar and assessment of cure. J Clin Microbiol. 2005;43(7):3038–41. doi: 10.1128/JCM.43.7.3038-3041.2005 16000412 PMC1169152

[23] Srivastava P, Mehrotra S, Tiwary P, Chakravarty J, Sundar S. Diagnosis of indian visceral leishmaniasis by nucleic acid detection using pcr. PLoS One. 2011;6(4):4–8. doi: 10.1371/journal.pone.0019304 21559398 PMC3084819

[24] Zijlstra EE, Daifalla NS, Kager PA, Khalil EAG, El-Hassan AM, Reed SG, et al. rK39 enzyme-linked immunosorbent assay for diagnosis of Leishmania donovani infection. Clin Diagn Lab Immunol. 1998;5(5):717–20. doi: 10.1128/CDLI.5.5.717-720.1998 9729541 PMC95645

[25] Caballero ZC, Sousa OE, Marques WP, Saez-Alquezar A, Umezawa ES. Evaluation of serological tests to identify Trypanosoma cruzi infection in humans and determine cross-reactivity with Trypanosoma rangeli and Leishmania spp. Clin Vaccine Immunol. 2007;14(8):1045–9. doi: 10.1128/CVI.00127-07 17522327 PMC2044488

[26] Harith AE, Kolk AH, Kager PA, Leeuwenburg J, Muigai R, Kiugu S, et al. A simple and economical direct agglutination test for serodiagnosis and sero-epidemiological studies of visceral leishmaniasis. Trans R Soc Trop Med Hyg. 1986;80(4):583–6. doi: 10.1016/0035-9203(86)90149-5 3101241

[27] Singh D, Pandey K, Das VNR, Das S, Verma N, Ranjan A, et al. Evaluation of rK-39 strip test using urine for diagnosis of visceral leishmaniasis in an endemic region of India. Am J Trop Med Hyg. 2013;88(2):222–6. doi: 10.4269/ajtmh.2012.12-0489 23149580 PMC3583308

[28] Eyayu T, Yasin M, Workineh L, Tiruneh T, Andualem H, Sema M, et al. Evaluation of urine sample for diagnosis of visceral leishmaniasis using rK-39 immunochromatographic test in Northwest Ethiopia. PLoS One [Internet]. 2022;17(2 February):1–12. Available from: doi: 10.1371/journal.pone.0263696 35130316 PMC8820633

[29] Singh S. New developments in diagnosis of leishmaniasis. Indian J Med Res. 2006;123(3):311–30. 16778313

[30] Adhya S, Chatterjee M, Hassan MQ, Mukherjee S, Sen S. Detection of Leishmania in the blood of early kala-azar patients with the aid of the polymerase chain reaction. Trans R Soc Trop Med Hyg [Internet]. 1995;89(6):622–4. Available from: http://www.sciencedirect.com/science/article/pii/0035920395904166 doi: 10.1016/0035-9203(95)90416-6 8594675

[31] Cascio A, Calattini S, Colomba C, Scalamogna C, Galazzi M, Pizzuto M, et al. Polymerase chain reaction in the diagnosis and prognosis of Mediterranean visceral leishmaniasis in immunocompetent children. Pediatrics. 2002 Feb;109(2):E27. doi: 10.1542/peds.109.2.e27 11826237

[32] Verma S, Kumar R, Katara GK, Singh LC, Negi NS, Ramesh V, et al. Quantification of parasite load in clinical samples of leishmaniasis patients: Il-10 level correlates with parasite load in visceral leishmaniasis. PLoS One. 2010;5(4). doi: 10.1371/journal.pone.0010107 20404924 PMC2852412

[33] Ghasemian M, Gharavi MJ, Akhlaghi L, Mohebali M, Meamar AR, Aryan E, et al. SYBR green-based detection of Leishmania infantum DNA using peripheral blood samples. J Parasit Dis [Internet]. 2016;40(1):81–7. Available from: doi: 10.1007/s12639-014-0452-4 27065603 PMC4815842

[34] Hossain F, Ghosh P, Anik Ashfaq Khan M, Duthie MS, Vallur AC, Picone A, et al. Real-time PCR in detection and quantitation of Leishmania donovani for the diagnosis of visceral leishmaniasis patients and the monitoring of their response to treatment. PLoS One. 2017;12(9):1–16. doi: 10.1371/journal.pone.0185606 28957391 PMC5619796

[35] Islam MZ, Itoh M, Islam MAU, Ekram ARMS, Rahman MA, Takagi H, et al. ELISA with recombinant rKRP42 antigen using urine samples: A tool for predicting clinical visceral leishmaniasis cases and its outbreak. Am J Trop Med Hyg. 2012;87(4):658–62. doi: 10.4269/ajtmh.2012.12-0168 22869633 PMC3516315

[36] Islam MZ, Itoh M, Mirza R, Ahmed I, Ekram ARMS, Sarder AH, et al. Direct agglutination test with urine samples for the diagnosis of visceral leishmaniasis. Am J Trop Med Hyg. 2004;70(1):78–82. 14971702

[37] Rao RU, Atkinson LJ, Ramzy RMR, Helmy H, Farid HA, Bockarie MJ, et al. A real-time PCR-based assay for detection of Wuchereria bancrofti DNA in blood and mosquitoes. Am J Trop Med Hyg. 2006;74(5):826–32. 16687688 PMC2196401

[38] Galluzzi L, Ceccarelli M, Diotallevi A, Menotta M, Magnani M. Real-time PCR applications for diagnosis of leishmaniasis. Parasites and Vectors. 2018;11(1):1–13. 29716641 10.1186/s13071-018-2859-8 PMC5930967

[39] Goto H, Prianti MDG. Immunoactivation and immunopathogeny during active visceral leishmaniasis. Rev Inst Med Trop Sao Paulo. 2009;51(5):241–6. doi: 10.1590/s0036-46652009000500002 19893975

[40] World Health Organization. Ending the neglect to attain the Sustainable Development Goals A road map for neglected tropical diseases 2021–2030 [Internet]. World Health Organization; 2020. 196 p. Available from: https://www.who.int/publications/i/item/9789240010352

