# Supplementary figures and images for: Real Time PCR-based diagnosis of human visceral leishmaniasis using urine samples
Source: PLOS Glob Public Health. 2022 Dec 29;2(12):e0000834. doi: 10.1371/journal.pgph.0000834 (PMC10022223; doi:10.1371/journal.pgph.0000834)

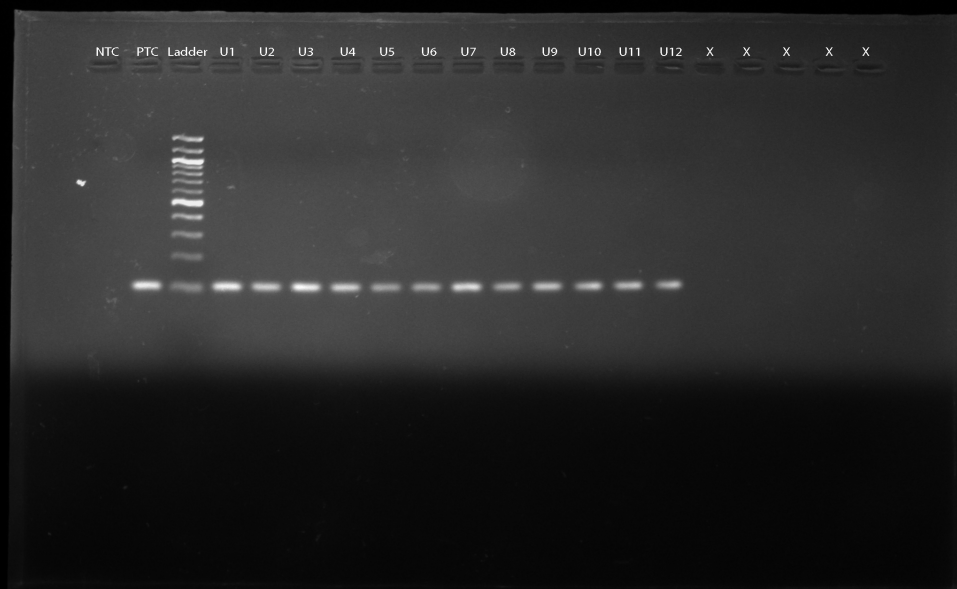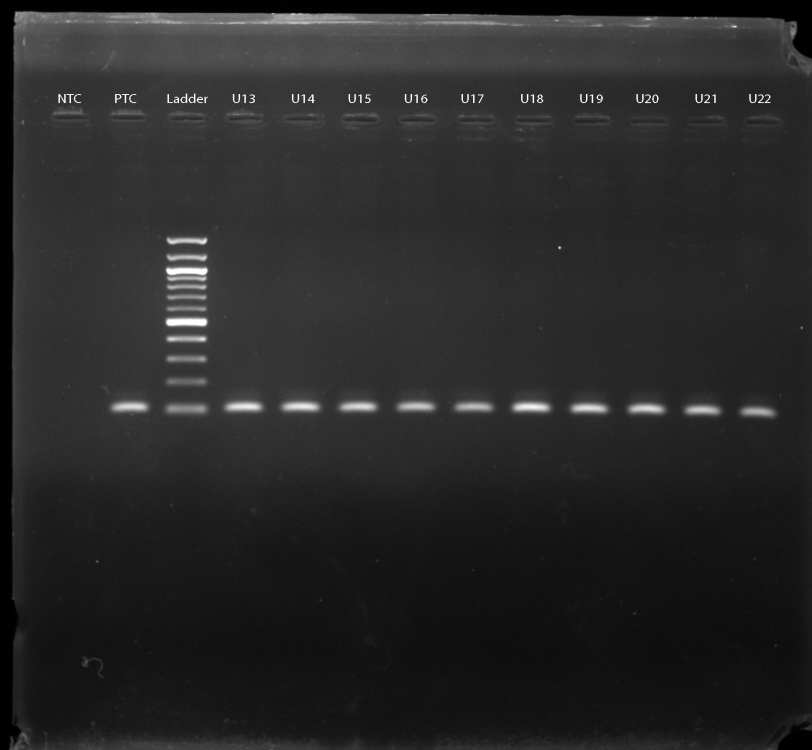

Supplement: S1 Fig — (PDF) [file pgph.0000834.s001.pdf]
