# Supplementary material for: Real Time PCR-based diagnosis of human visceral leishmaniasis using urine samples
Source: PLOS Glob Public Health. 2022 Dec 29;2(12):e0000834. doi: 10.1371/journal.pgph.0000834 (PMC10022223; doi:10.1371/journal.pgph.0000834)
Supplement: S1 Table — (DOCX) [file pgph.0000834.s002.docx]

| Parasite Load  (parasite/µL) | Intra assay Variation of Ct value | | | | |
| --- | --- | --- | --- | --- | --- |
|  | Replicate 1 | Replicate 2 | Replicate  3 | Mean±SD | CV% |
| 1x10^4^ | 9.26 | 9.21 | 9.22 | 9.23±0.026 | 0.29% |
| 1x10^3^ | 12.96 | 13.02 | 12.98 | 12.99±0.025 | 0.18% |
| 1x10^2^ | 17.11 | 17.15 | 17.18 | 17.16±0.03 | 0.17% |
| 1x10^1^ | 19.98 | 20.04 | 19.94 | 19.99±0.041 | 0.21% |
| 1x10^0^ | 23.19 | 23.21 | 22.95 | 23.12±0.12 | 0.51% |
| 1x10^-1^ | 28.63 | 27.52 | 27.82 | 27.99±0.46 | 1.67% |

**Supporting information**

**S1 Table:** Reproducibility of Real time PCR based diagnosis of VL (Assay 2)
