# Supplementary material for: Real Time PCR-based diagnosis of human visceral leishmaniasis using urine samples
Source: PLOS Glob Public Health. 2022 Dec 29;2(12):e0000834. doi: 10.1371/journal.pgph.0000834 (PMC10022223; doi:10.1371/journal.pgph.0000834)
Supplement: S2 Table — (DOCX) [file pgph.0000834.s003.docx]

**Supporting information**

**S2 Table: Limit of detection of parasite body in urine and blood**

| Parasite Load in urine (parasite/µL) | Intra assay Variation of Ct value | | | | Variation of Ct value with reproducibility assay | | | | Limit of detection |
| --- | --- | --- | --- | --- | --- | --- | --- | --- | --- |
|  | Replicate 1 | Replicate 2 | Mean±SD | CV% | Mean Ct value from  reproducibility assay | Mean Ct value from  Urine spiked assay | Mean±SD | CV% |  |
| 1x10^2^ | 17.29 | 17.15 | 17.22±0.1 | 0.48% | 16.76 | 17.22 | 16.99±0.23 | 1.35% | 10fg DNA corresponding to 0.1 parasite in 1µL of urine |
| 1x10^1^ | 19.89 | 19.72 | 19.81±0.09 | 0.43% | 19.45 | 19.81 | 19.63±0.18 | 0.18% |  |
| 1x10^0^ | 24.11 | 23.99 | 24.05±0.085 | 0.25% | 23.53 | 24.05 | 23.79±0.26 | 1.09% |  |
| 1x10^-1^ | 28.62 | 29.91 | 29.26±0.65 | 2.2% | 28.03 | 29.26 | 28.64±0.87 | 3.04% |  |
|  |  |  |  |  |  |  |  |  |  |
| Parasite Load in blood buffy coat (parasite/µL) | Intra assay Variation of Ct value | | | | Variation of Ct value with reproducibility assay | | | | Limit of detection |
|  | Replicate 1 | Replicate 2 | Mean±SD | CV% | Mean Ct value from  reproducibility assay | Mean Ct value from  Blood buffy coat spiked assay | Mean±SD | CV% |  |
| 1x10^2^ | 16.59 | 16.75 | 16.72±0.03 | 0.18% | 16.76 | 16.72 | 16.74±0.02 | 0.12% | 10fg DNA corresponding to 0.1 parasite in 1µL of Blood buffy coat |
| 1x10^1^ | 19.09 | 18.99 | 19.4±0.05 | 0.26% | 19.45 | 19.4 | 19.42±0.03 | 0.17% |  |
| 1x10^0^ | 23.71 | 23.89 | 23.8±0.09 | 0.38% | 23.53 | 23.80 | 23.67±0.14 | 0.60% |  |
| 1x10^-1^ | 27.22 | 26.81 | 27.01±0.21 | 0.76% | 28.03 | 27.01 | 27.53±0.51 | 1.9% |  |
