# Supplementary material for: Real Time PCR-based diagnosis of human visceral leishmaniasis using urine samples
Source: PLOS Glob Public Health. 2022 Dec 29;2(12):e0000834. doi: 10.1371/journal.pgph.0000834 (PMC10022223; doi:10.1371/journal.pgph.0000834)
Supplement: S3 Table — (DOCX) [file pgph.0000834.s004.docx]

**Supporting information**

**S3 Table: Result of conventional-PCR and Real time PCR based diagnosis of VL using urine DNA of CDVL patients.**

| SL of CDVL participants | Conventional-PCR | Real time PCR | | |
| --- | --- | --- | --- | --- |
|  |  | Ct | Parasites/mL urine | Tm value |
| 1 | Positive | 25.44175 | 7.061560202 | 81.51783 |
| 2 | Positive | 26.67978 | 2.77298917 | 81.07362 |
| 3 | Positive | 24.9577 | 10.17704327 | 80.18032 |
| 4 | Positive | 26.35063 | 3.555307028 | 81.36894 |
| 5 | Positive | 27.54816 | 1.439476731 | 81.22006 |
| 6 | Positive | 28.29082 | 0.82164641 | 81.07117 |
| 7 | Positive | 25.57795 | 6.371481345 | 81.07117 |
| 8 | Positive | 23.44175 | 31.96720255 | 81.07117 |
| 9 | Positive | 27.67978 | 1.303305386 | 81.22006 |
| 10 | Positive | 25.9577 | 4.783212085 | 80.92229 |
| 11 | Positive | 20.35063 | 329.829132 | 81.22006 |
| 12 | Positive | 24.54816 | 13.86470391 | 81.22006 |
| 13 | Positive | 27.0082 | 1.439476731 | 81.22006 |
| 14 | Positive | 26.795 | 2.541951774 | 80.92229 |
| 15 | Positive | 26.24175 | 3.859926893 | 80.77341 |
| 16 | Positive | 23.64278 | 27.46543597 | 81.22006 |
| 17 | Positive | 26.9577 | 2.248110501 | 81.22006 |
| 18 | Positive | 22.35063 | 72.85930849 | 81.07117 |
| 19 | Positive | 23.54816 | 29.49935925 | 81.07117 |
| 20 | Positive | 27.29082 | 1.748183213 | 81.36894 |
| 21 | Positive | 26.57795 | 2.994597326 | 81.22006 |
| 22 | Positive | 27.13323 | 1.969069381 | 81.22006 |
| 23 | Positive | 22.31312 | 74.9522455 | 81.22006 |
