# Supplementary material for: Real Time PCR-based diagnosis of human visceral leishmaniasis using urine samples
Source: PLOS Glob Public Health. 2022 Dec 29;2(12):e0000834. doi: 10.1371/journal.pgph.0000834 (PMC10022223; doi:10.1371/journal.pgph.0000834)
Supplement: S4 Table — (DOCX) [file pgph.0000834.s005.docx]

**Supporting information**

**S4 Table: Result of conventional-PCR and Real time PCR based diagnosis of VL using blood buffy coat DNA of CDVL patients.**

| SL of CDVL participants | Conventional-PCR | Real time PCR | | |
| --- | --- | --- | --- | --- |
|  |  | Ct | Parasites/mL blood | Tm value |
| 1 | Positive | 20.90433 | 1085.674627 | 80.61872 |
| 2 | Positive | 24.13635 | 94.60496285 | 80.94589 |
| 3 | Positive | 18.29638 | 3847.604586 | 80.91451 |
| 4 | Positive | 22.51458 | 321.8811314 | 81.54244 |
| 5 | Positive | 24.59305 | 67.01331216 | 81. 54244 |
| 6 | Positive | 24.69993 | 61.81799565 | 81.52452 |
| 7 | Positive | 20.46497 | 1512.748404 | 81.52452 |
| 8 | Positive | 24.99496 | 49.47365515 | 81.73652 |
| 9 | Positive | 21.06259 | 963.3983987 | 81.13492 |
| 10 | Positive | 20.35961 | 1638.002108 | 80.25632 |
| 11 | Positive | 18.17459 | 4007.604586 | 81.73652 |
| 12 | Positive | 20.58422 | 1382.497619 | 81.25234 |
| 13 | Positive | 21.71618 | 588.1559065 | 81.22006 |
| 14 | Positive | 19.9105 | 2299.209346 | 80.52432 |
| 15 | Positive | 20.32091 | 1686.569581 | 80.87245 |
| 16 | Positive | 20.42031 | 1564.626948 | 80.87245 |
| 17 | Positive | 20.59168 | 1374.732637 | 81.24522 |
| 18 | Positive | 19.79551 | 2507.748419 | 81.02452 |
| 19 | Positive | 22.97402 | 227.5329156 | 81.24522 |
| 20 | Positive | 18.86803 | 5051.338785 | 81.32452 |
| 21 | Positive | 23.98985 | 105.6699641 | 81.22345 |
| 22 | Positive | 20.46974 | 1507.310106 | 81.22345 |
| 23 | Positive | 18.04743 | 5532.882866 | 81.25455 |
