# Supplementary material for: Real Time PCR-based diagnosis of human visceral leishmaniasis using urine samples
Source: PLOS Glob Public Health. 2022 Dec 29;2(12):e0000834. doi: 10.1371/journal.pgph.0000834 (PMC10022223; doi:10.1371/journal.pgph.0000834)
Supplement: S5 Table — (DOCX) [file pgph.0000834.s006.docx]

**Supporting information**

**S5 Table: Result of Real time PCR bone marrow DNA of CVL patients.**

| SL of CVL participants | Real time PCR | |
| --- | --- | --- |
|  | Ct | Tm value |
| 1 | 11.25332 | 80.91245 |
| 2 | 12.514085 | 80.94234 |
| 3 | 12.7176 | 80.82452 |
| 4 | 13.62334 | 81.15345 |
| 5 | 16.8493 | 81. 76452 |
| 6 | 9.009938 | 81.52522 |
| 7 | 14.2695 | 81.52522 |
| 8 | 12.43015 | 81.63522 |
| 9 | 15.89904 | 81.16352 |
| 10 | 13.52783 | 80.22555 |
| 11 | 20.91084 | 81.25245 |
| 12 | 19.96744 | 81.22525 |
| 13 | 14.2695 | 81.24722 |
| 14 | 13.72668 | 80.51345 |
| 15 | 15.87666 | 80.87353 |
| 16 | 19.27126 | 80.25245 |
| 17 | 17.90009 | 81.22525 |
| 18 | 20.17348 | 81.12635 |
| 19 | 19.73004 | 81.22356 |
