# Supplementary material for: Real Time PCR-based diagnosis of human visceral leishmaniasis using urine samples
Source: PLOS Glob Public Health. 2022 Dec 29;2(12):e0000834. doi: 10.1371/journal.pgph.0000834 (PMC10022223; doi:10.1371/journal.pgph.0000834)
Supplement: S6 Table — (DOCX) [file pgph.0000834.s007.docx]

**Supporting information**

**S6 Table: Result of conventional-PCR and Real time PCR based diagnosis of VL using DNA of Control participants.**

***Healthy control (HC)**

***Disease control (DC)**

| SL | ID of Control participants | Control Type | Sample Type | Conventional-PCR | Real time PCR | | |
| --- | --- | --- | --- | --- | --- | --- | --- |
|  |  |  |  |  | Ct | Parasites/mL sample | Tm value |
| 1 | HC1 | Non endemic | Urine | Negative | N/A | N/A | N/A |
| 2 | HC2 | Non endemic | Urine | Negative | N/A | N/A | N/A |
| 3 | HC3 | Non endemic | Urine | Negative | N/A | N/A | N/A |
| 4 | HC4 | Non endemic | Urine | Negative | N/A | N/A | N/A |
| 5 | HC5 | Non endemic | Urine | Negative | N/A | N/A | N/A |
| 6 | HC6 | Non endemic | Urine | Negative | N/A | N/A | N/A |
| 7 | HC7 | Non endemic | Urine | Negative | N/A | N/A | N/A |
| 8 | HC8 | Non endemic | Urine | Negative | N/A | N/A | N/A |
| 9 | HC9 | Non endemic | Urine | Negative | N/A | N/A | N/A |
| 10 | HC10 | Non endemic | Urine | Negative | N/A | N/A | N/A |
| 11 | HC11 | Endemic | Urine | Negative | N/A | N/A | N/A |
| 12 | HC12 | Endemic | Urine | Negative | N/A | N/A | N/A |
| 13 | HC13 | Endemic | Urine | Negative | N/A | N/A | N/A |
| 14 | HC14 | Endemic | Urine | Negative | N/A | N/A | N/A |
| 15 | HC15 | Endemic | Urine | Negative | N/A | N/A | N/A |
| 16 | HC16 | Endemic | Urine | Negative | N/A | N/A | N/A |
| 17 | HC17 | Endemic | Urine | Negative | N/A | N/A | N/A |
| 18 | HC18 | Endemic | Urine | Negative | N/A | N/A | N/A |
| 19 | HC19 | Endemic | Urine | Negative | N/A | N/A | N/A |
| 20 | HC20 | Endemic | Urine | Negative | N/A | N/A | N/A |
| 21 | HC1 | Non endemic | Blood buffy coat | Negative | N/A | N/A | N/A |
| 22 | HC2 | Non endemic | Blood buffy coat | Negative | N/A | N/A | N/A |
| 23 | HC3 | Non endemic | Blood buffy coat | Negative | N/A | N/A | N/A |
| 24 | HC4 | Non endemic | Blood buffy coat | Negative | N/A | N/A | N/A |
| 25 | HC5 | Non endemic | Blood buffy coat | Negative | N/A | N/A | N/A |
| 26 | HC6 | Non endemic | Blood buffy coat | Negative | N/A | N/A | N/A |
| 27 | HC7 | Non endemic | Blood buffy coat | Negative | N/A | N/A | N/A |
| 28 | HC8 | Non endemic | Blood buffy coat | Negative | N/A | N/A | N/A |
| 29 | HC9 | Non endemic | Blood buffy coat | Negative | N/A | N/A | N/A |
| 30 | HC10 | Non endemic | Blood buffy coat | Negative | N/A | N/A | N/A |
| 31 | HC11 | Endemic | Blood buffy coat | Negative | N/A | N/A | N/A |
| 32 | HC12 | Endemic | Blood buffy coat | Negative | N/A | N/A | N/A |
| 33 | HC13 | Endemic | Blood buffy coat | Negative | N/A | N/A | N/A |
| 34 | HC14 | Endemic | Blood buffy coat | Negative | N/A | N/A | N/A |
| 35 | HC15 | Endemic | Blood buffy coat | Negative | N/A | N/A | N/A |
| 36 | HC16 | Endemic | Blood buffy coat | Negative | N/A | N/A | N/A |
| 37 | HC17 | Endemic | Blood buffy coat | Negative | N/A | N/A | N/A |
| 38 | HC18 | Endemic | Blood buffy coat | Negative | N/A | N/A | N/A |
| 39 | HC19 | Endemic | Blood buffy coat | Negative | N/A | N/A | N/A |
| 40 | HC20 | Endemic | Blood buffy coat | Negative | N/A | N/A | N/A |
| 41 | DC1 | Tuberculosis | Blood | Negative | N/A | N/A | N/A |
| 42 | DC 2 | Tuberculosis | Blood | Negative | N/A | N/A | N/A |
| 43 | DC 3 | Tuberculosis | Blood | Negative | N/A | N/A | N/A |
| 44 | DC 4 | Tuberculosis | Blood | Negative | N/A | N/A | N/A |
| 45 | DC 5 | Tuberculosis | Blood | Negative | N/A | N/A | N/A |
| 46 | DC 6 | Tuberculosis | Blood | Negative | N/A | N/A | N/A |
| 47 | DC 7 | Tuberculosis | Blood | Negative | N/A | N/A | N/A |
| 48 | DC 8 | Tuberculosis | Blood | Negative | N/A | N/A | N/A |
| 49 | DC 9 | Tuberculosis | Blood | Negative | N/A | N/A | N/A |
| 50 | DC 10 | Tuberculosis | Blood | Negative | N/A | N/A | N/A |
| 51 | DC 11 | Malaria | Blood | Negative | N/A | N/A | N/A |
| 52 | DC 12 | Malaria | Blood | Negative | N/A | N/A | N/A |
| 53 | DC 13 | Malaria | Blood | Negative | N/A | N/A | N/A |
| 54 | DC 14 | Dengue | Blood | Negative | N/A | N/A | N/A |
| 55 | DC 15 | Dengue | Blood | Negative | N/A | N/A | N/A |
| 56 | DC 16 | Dengue | Blood | Negative | N/A | N/A | N/A |
| 57 | DC 17 | Dengue | Blood | Negative | N/A | N/A | N/A |
| 58 | DC 18 | Dengue | Blood | Negative | N/A | N/A | N/A |
| 59 | DC 19 | Dengue | Blood | Negative | N/A | N/A | N/A |
| 60 | DC 20 | Dengue | Blood | Negative | N/A | N/A | N/A |
| 61 | DC 21 | Dengue | Blood | Negative | N/A | N/A | N/A |
| 62 | DC 22 | Dengue | Blood | Negative | N/A | N/A | N/A |
| 63 | DC 23 | Dengue | Blood | Negative | N/A | N/A | N/A |
| 64 | DC 24 | Dengue | Blood | Negative | N/A | N/A | N/A |
| 65 | DC 25 | Dengue | Blood | Negative | N/A | N/A | N/A |
